# Expression characteristics of lipid metabolism-related genes and correlative immune infiltration landscape in acute myocardial infarction

**DOI:** 10.1038/s41598-024-65022-3

**Published:** 2024-06-18

**Authors:** Jiahe Wu, Jingyi Luo, Huanhuan Cai, Haoyan Zhu, Zhe Lei, Yi Lu, Xinchen Gao, Lihua Ni, Zhibing Lu, Xiaorong Hu

**Affiliations:** 1 Department of Cardiology, Zhongnan Hospital of Wuhan University, No. 169 Donghu Road, Wuchang District, Wuhan, 430071 China; 2 Institute of Myocardial Injury and Repair, Wuhan University, Wuhan, China; 3 Department of Stomatology, Zhongnan Hospital of Wuhan University, Wuhan, China; 4 Department of Nephrology, Zhongnan Hospital of Wuhan University, No. 169 Donghu Road, Wuchang District, Wuhan, 430071 China

**Keywords:** Acute myocardial infarction, Lipid metabolism, Gene expression pattern, Immune infiltration, Biomarkers, Data mining, Gene expression, Acute coronary syndromes

## Abstract

Lipid metabolism is an important part of the heart's energy supply. The expression pattern and molecular mechanism of lipid metabolism-related genes (LMRGs) in acute myocardial infarction (AMI) are still unclear, and the link between lipid metabolism and immunity is far from being elucidated. In this study, 23 Common differentially expressed LMRGs were discovered in the AMI-related mRNA microarray datasets GSE61144 and GSE60993. These genes were mainly related to “leukotriene production involved in inflammatory response”, “lipoxygenase pathway”, “metabolic pathways”, and “regulation of lipolysis in adipocytes” pathways. 12 LMRGs (*ACSL1*, *ADCY4*, *ALOX5*, *ALOX5AP*, *CCL5*, *CEBPB*, *CEBPD*, *CREB5*, *GAB2*, *PISD*, *RARRES3*, and *ZNF467*) were significantly differentially expressed in the validation dataset GSE62646 with their AUC > 0.7 except for *ALOX5AP* (AUC = 0.699). Immune infiltration analysis and Pearson correlation analysis explored the immune characteristics of AMI, as well as the relationship between these identified LMRGs and immune response. Lastly, the up-regulation of *ACSL1*, *ALOX5AP*, *CEBPB*, and *GAB2* was confirmed in the mouse AMI model. Taken together, LMRGs *ACSL1*, *ALOX5AP*, *CEBPB*, and *GAB2* are significantly upregulated in AMI patients' blood, peripheral blood of AMI mice, myocardial tissue of AMI mice, and therefore might be new potential biomarkers for AMI.

## Introduction

Acute myocardial infarction (AMI) is known for its high disability and mortality, which seriously endangers human health^1^. AMI is the result of acute interruption of myocardial blood flow followed by myocardial ischemic hypoxic necrosis^2^. Generally speaking, the traditional risk factors include age, smoking, hypertension, obesity, diabetes, family history, etc.^3^. Early opening of the infarcted vessel and restoration of myocardial blood perfusion, namely drug thrombolysis or percutaneous coronary intervention (PCI) surgery, are the main strategies to reduce the size of myocardial infarction, reduce mortality and improve the prognosis^4,5^. Clinically, ECG and high-sensitive cardiac troponin I are widely used as the diagnostic indicators of AMI. However, about one-third of patients still do not receive reperfusion therapy as early as possible due to the belated diagnosis^6^. Therefore, searching for potential biomarkers with high sensitivity and specificity at the beginning of AMI is of great value for the individualized diagnosis and treatment of AMI patients.

Lipid metabolism is closely related to AMI. Under physiological conditions, 50–70 percent of the heart's energy support comes from fatty acid β-oxidation and the rest comes from the metabolism of glucose, lactate, ketone bodies, and amino acids^7^. During AMI, the blood supply to the heart is insufficient, oxygen and nutrients are not available, and the metabolic pattern of the heart is correspondingly altered^8,9^. There have been some researches on gene regulation or molecular therapy related to lipid metabolism in AMI. Higher lipid availability has been reported to promote ischemia-induced cardiac dysfunction and reduce myocardial mitochondrial efficiency^10^. *PEDF* (Cytochrome c-550 PedF) promotes TG (Triglycerides) degradation of cardiomyocytes through *ATGL* (Patatin like phospholipase domain containing 2), reduces infarct size, and protects cardiac function^11^. *APOE* (Apolipoprotein E) deficiency leads to the formation of excess neutrophil extracellular trap and aggravates myocardial injury in mice model of myocardial infarction^12^. Omega-3 polyunsaturated fatty acid supplementation reduced the levels of apolipoprotein B, triglycerides, lipoprotein (a), and exerted a protective effect on AMI by affecting the systemic eicosanoid metabolic status^13^. These studies indicate that lipid metabolism is associated with the formation and development of AMI, and that intervention targeting this process may be an effective therapeutic strategy.

**Figure 1:**
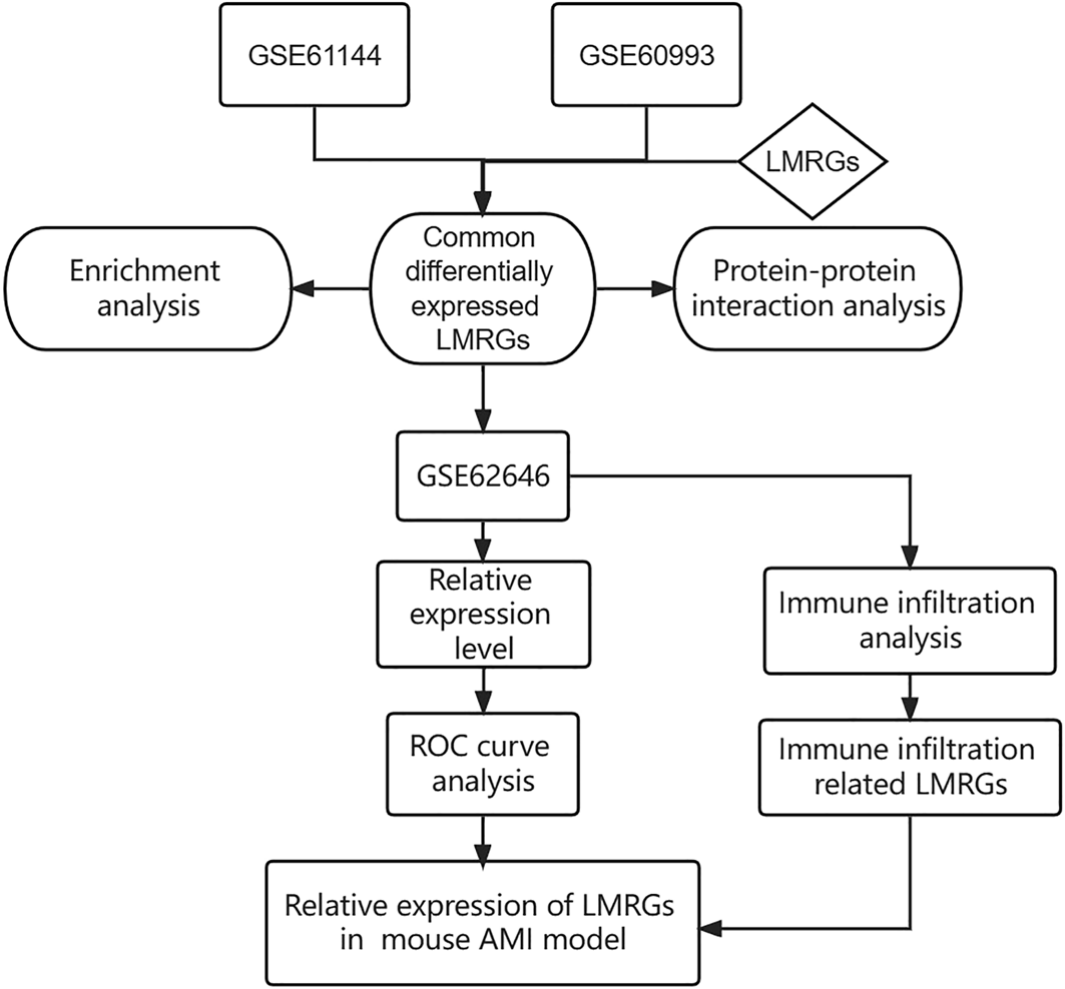
The workflow of the specific analysis.

The expression pattern and molecular mechanism of lipid metabolism-related genes (LMRGs) in AMI remain unclear, and their relationship with immunity is far from clarified. This study used bioinformatics methods to screen and verify differentially expressed LMRGs based on AMI-related datasets. ROC analysis was performed to evaluate the diagnostic value of these genes. Immune infiltration analysis and Pearson correlation analysis were performed to explore the relationship between these genes and immunity. Finally, the expression of the identified LMRGs was verified in the mouse AMI model. Figure 1 shows the workflow of the specific analysis. Above all, this study will explore the mechanism of lipid metabolism involved in AMI and provide new directions for the clinical individualized diagnosis and treatment of AMI.

## Results

### Identification of DEGs and differentially expressed LMRGs in *AMI*

**Figure 2:**
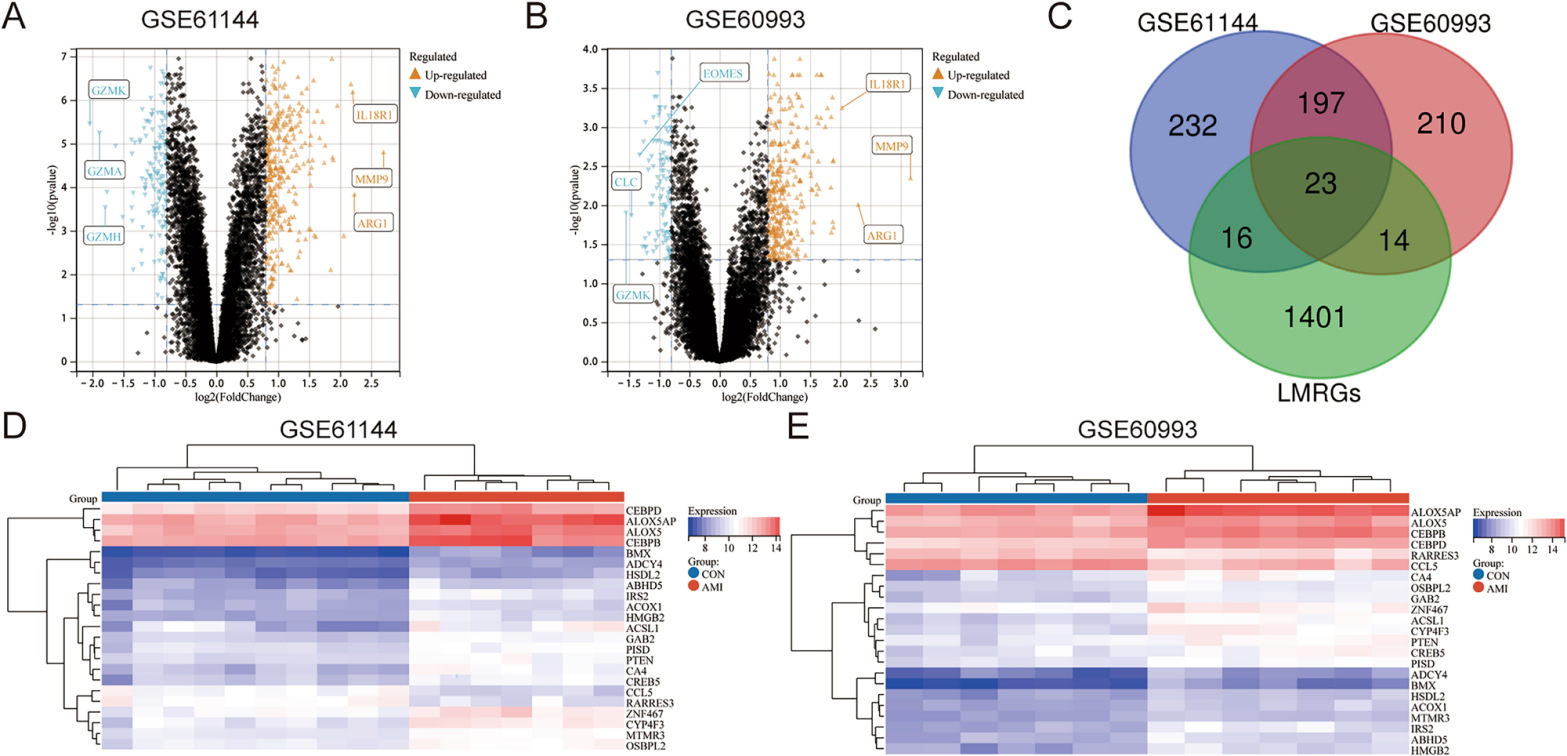
Identification of DEGs and differentially expressed LMRGs in AMI. (**A**) The volcano plot of GSE61144. (**B**) The volcano plot of GSE60993. The top three up-regulated or down-regulated gene names are shown in panel **A**,**B**. The horizontal lines represent the filtering thresholds for *P*-values. The vertical lines represent the screening threshold for the fold changes. (**C**) Venn diagram of DEGs in GSE61144, GSE60993, and candidate LMRGs. (**D**) Heatmap of common differentially expressed LMRGs in GSE61144. (**E**) Heatmap of common differentially expressed LMRGs in GSE60993.

Differential expression analysis was performed on datasets GSE61144 and GSE60993 to compare the differences in gene expression between patients with AMI and controls. With “*P* value < 0.05 and |logFC|≥ 0.8” as the threshold, 468 DEGs were identified in GSE61144, of which 325 were high-expressed and 143 were low-expressed; 444 DEGs were identified in GSE60993, of which 339 were high-expressed and 105 were low-expressed. Supplementary File 1 presents the details of the DEGs in these two datasets. Figure 2A,B shows the volcano plot of the differential expression analysis for GSE61144 and GSE60993. The DEGs in these two datasets were intersected with 1454 candidate LMRGs obtained from the literature search mentioned above, 23 common differentially expressed LMRGs were identified (Fig. 2C). They were *ABHD5* (Abhydrolase domain containing 5, lysophosphatidic acid acyltransferase), *ACOX1* (Acyl-CoA oxidase 1), *ACSL1* (Acyl-CoA synthetase long chain family member 1), *ADCY4* (Adenylate cyclase 4), *ALOX5* (Arachidonate 5-lipoxygenase), *ALOX5AP* (Arachidonate 5-lipoxygenase activating protein), *BMX* (BMX non-receptor tyrosine kinase), *CA4* (Carbonic anhydrase 4), *CCL5* (C–C motif chemokine ligand 5), *CEBPB* (CCAAT enhancer binding protein beta), *CEBPD* (CCAAT enhancer binding protein delta), *CREB5* (cAMP responsive element binding protein 5), *CYP4F3* (Cytochrome P450 family 4 subfamily F member 3), *GAB2* (GRB2 associated binding protein 2), *HMGB2* (High mobility group box 2), *HSDL2* (Hydroxysteroid dehydrogenase like 2), *IRS2* (Insulin receptor substrate 2), *MTMR3* (Myotubularin related protein 3), *OSBPL2* (Oxysterol binding protein like 2), *PISD* (Phosphatidylserine decarboxylase), *PTEN* (Phosphatase and tensin homolog), *RARRES3* (Phospholipase A and acyltransferase 4), and *ZNF467* (Zinc finger protein 467). After the Z-Score transformation of the raw gene expression data, we further performed cluster analysis using the Complete-linkage method. Row clustering groups the samples with the same expression characteristics. Column clustering brings together genes with consistent expression patterns. Figure 2D,E shows the heatmaps of these common differentially expressed LMRGs in GSE61144 and GSE60993.

### Expression pattern and molecular mechanism of Common differentially expressed LMRGs in *AMI*

**Figure 3:**
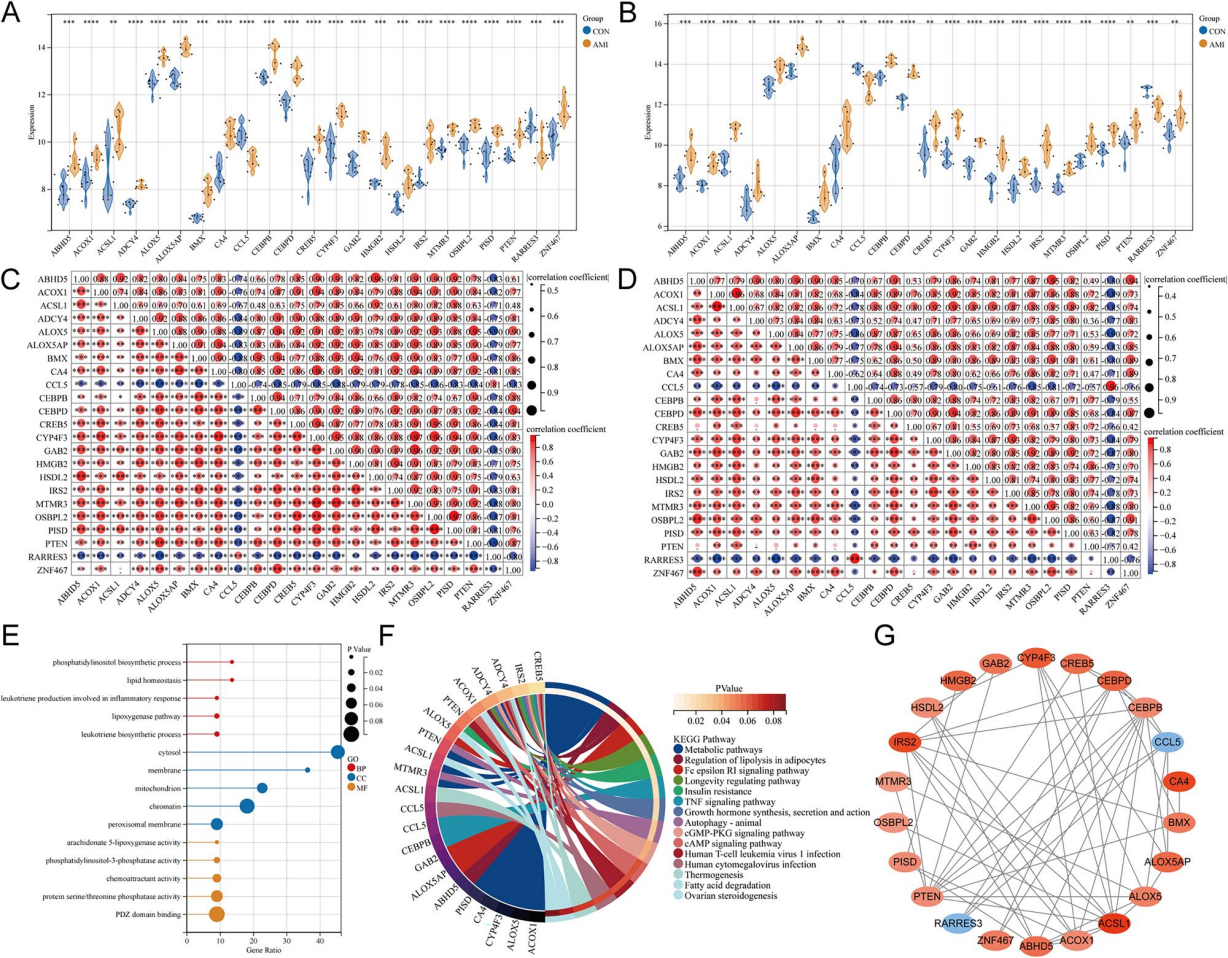
Expression pattern and molecular mechanism of Common differentially expressed LMRGs in AMI. (**A**) Expression pattern of common differentially expressed LMRGs in GSE61144 (n CON:n AMI = 10:7). (**B**) Expression pattern of common differentially expressed LMRGs in GSE60993 (n CON:n AMI = 7:7). (**C**) Correlation heatmap of common differentially expressed LMRGs in GSE61144. (**D**) Correlation heatmap of common differentially expressed LMRGs in GSE60993. (**E**) Lollipop plots of GO annotation results. (**F**) Circle plot of KEGG pathway enrichment analysis. The color blocks in the left half correspond to the gene names, the color blocks in the right half correspond to the signal pathways, the inner circle in the right half represents the *P*-value, and the thickness of the line represents the number of genes in the signal pathway. (**G**) The PPI Network of common differentially expressed LMRGs. For panels (**A**,**B**) **P < 0.01; ***P < 0.001; ****P < 0.0001.

The expression patterns of common differentially expressed LMRGs were compared based on their original gene expression data in GSE61144 and GSE60993. We used the online drawing tool of the Sangerbox website to draw the violin plots. As shown in Fig. 3A,B, the expression trend of these LMRGs was completely consistent in these two datasets, and their expression was up-regulated except for *CCL5* and *RARRES3*. The correlation heatmap demonstrated the correlation of the expression of these identified genes (Fig. 3C,D). The Pearson correlation analysis showed that *MTMR3* and *OSBPL2* had a strong positive correlation (GSE61144, correlation coefficient (CRC) = 0.93; GSE60993, CRC = 0.93), *GAB2* and *MTMR3* had a strong positive correlation (GSE61144, CRC = 0.96; GSE60993, CRC = 0.92), *ALOX5* and *CEBPD* had a strong positive correlation (GSE61144, CRC = 0.94; GSE60993, CRC = 0.87), but *ALOX5* and *RARRES3* had a strong negative correlation (GSE61144, CRC = − 0.9; GSE60993, CRC = − 0.9). Detailed Pearson correlation analysis of genes with strong correlations (CRC > 0.9) was presented in Supplementary File 2.

GO annotation and KEGG pathway enrichment analysis of these LMRGs were performed to explore the biological processes or signaling pathways involved in AMI. GO annotation results showed that these genes are mainly enriched in “phosphatidylinositol biosynthetic process”, “lipid homeostasis”, “leukotriene production involved in inflammatory response”, “lipoxygenase pathway”, “cytosol”, “membrane”, and “protein serine/threonine phosphatase activity” pathways (Fig. 3E). For KEGG pathway enrichment analysis, the mainly enriched pathways are “metabolic pathways”, “regulation of lipolysis in adipocytes”, “insulin resistance”, “TNF signaling pathway”, “autophagy—animal”, “cGMP-PKG signaling pathway”, “cAMP signaling pathway”, and “ovarian steroidogenesis” (Fig. 3F). PPI analysis of the proteins expressed by these LMRGs was performed by the String database. Finally, a PPI network with 22 nodes and 55 edges was constructed (Fig. 3G). In this network diagram, orange-red nodes represent high-expressed genes, blue nodes represent low-expressed gene genes, and the depth of color represents the degree of gene expression change in GSE61144.

### Validation of common differentially expressed LMRGs in external dataset GSE62646

**Figure 4:**
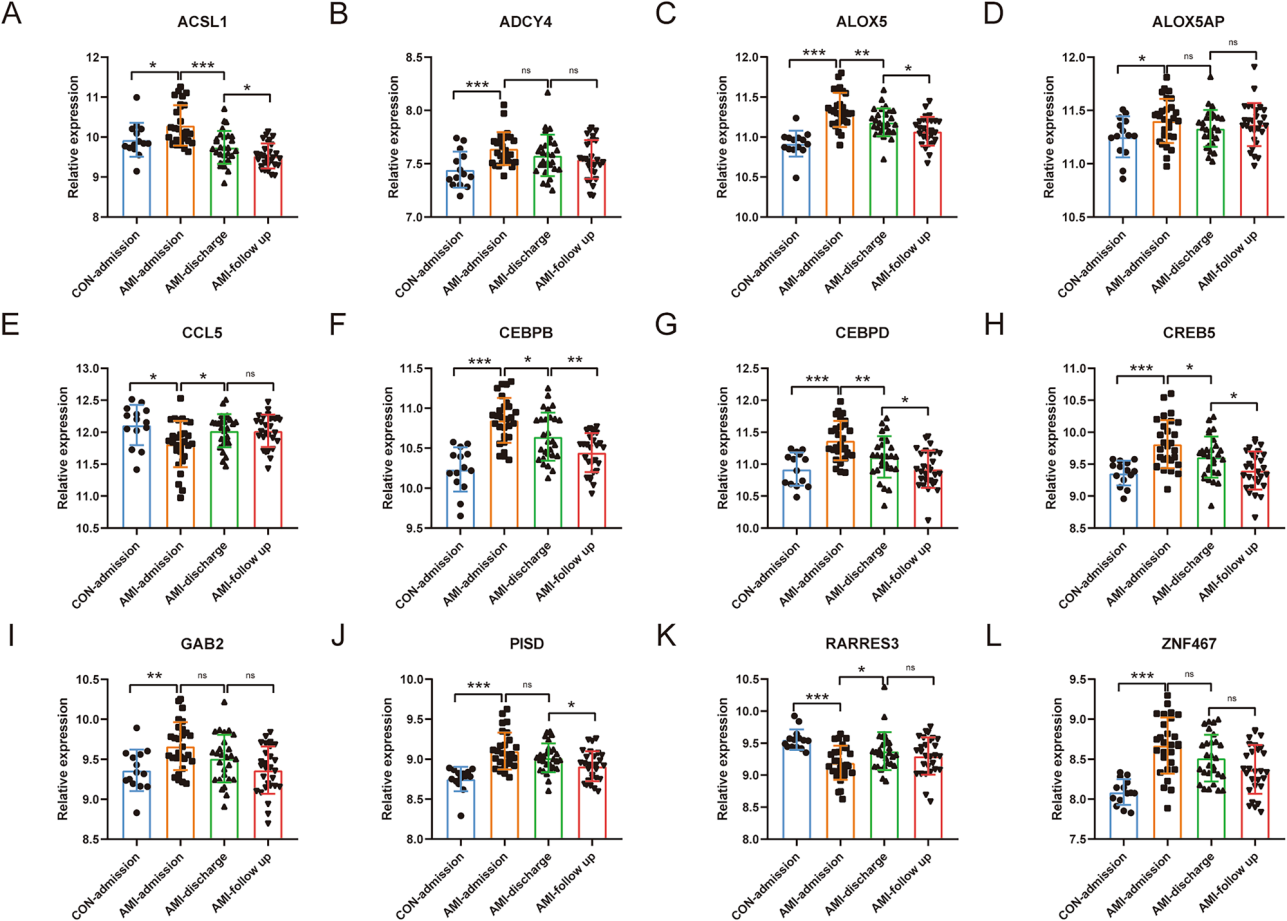
Validation of Common differentially expressed LMRGs in external dataset GSE62646. (**A**–**L**) Expression trends of *ACSL1*, *ADCY4*, *ALOX5*, *ALOX5AP*, *CCL5*, *CEBPB*, *CEBPD*, *CREB5*, *GAB2*, *PISD*, *RARRES3*, and *ZNF467* in dataset GSE62646 (n CON:n AMI on admission:n AMI at discharge:n AMI at follow-up = 14:28:28:28). For panels (**A**–**L**) ns P > 0.05; *P < 0.05; **P < 0.01; ***P < 0.001.

Based on the original gene expression data in GSE62646 (log2 transformed data), the expression of common differentially expressed LMRGs was evaluated. Compared with the control group (CON-admission), 12 LMRGs were significantly differentially expressed in patients with AMI (Fig. 4A–L). They were *ACSL1*, *ADCY4*, *ALOX5*, *ALOX5AP*, *CCL5*, *CEBPB*, *CEBPD*, *CREB5*, *GAB2*, *PISD*, *RARRES3*, and *ZNF467*. All of these genes except *CCL5* and *RARRES3* were significantly upregulated in AMI patients on admission (1st day of MI). Gene expression of *ACSL1*, *ALOX5*, *CEBPB*, *CEBPD*, and *CREB5* decreased significantly, while gene expression of *CCL5* and *RARRES3* increased significantly at discharge (4–6 days after MI). The expression levels of genes *ACSL1*, *ALOX5*, *CEBPB*, *CEBPD*, *CREB5*, and *PISD* were further decreased at follow-up (6 months after MI).

### ROC curve analysis of common differentially expressed LMRGs in external dataset GSE62646

**Figure 5:**
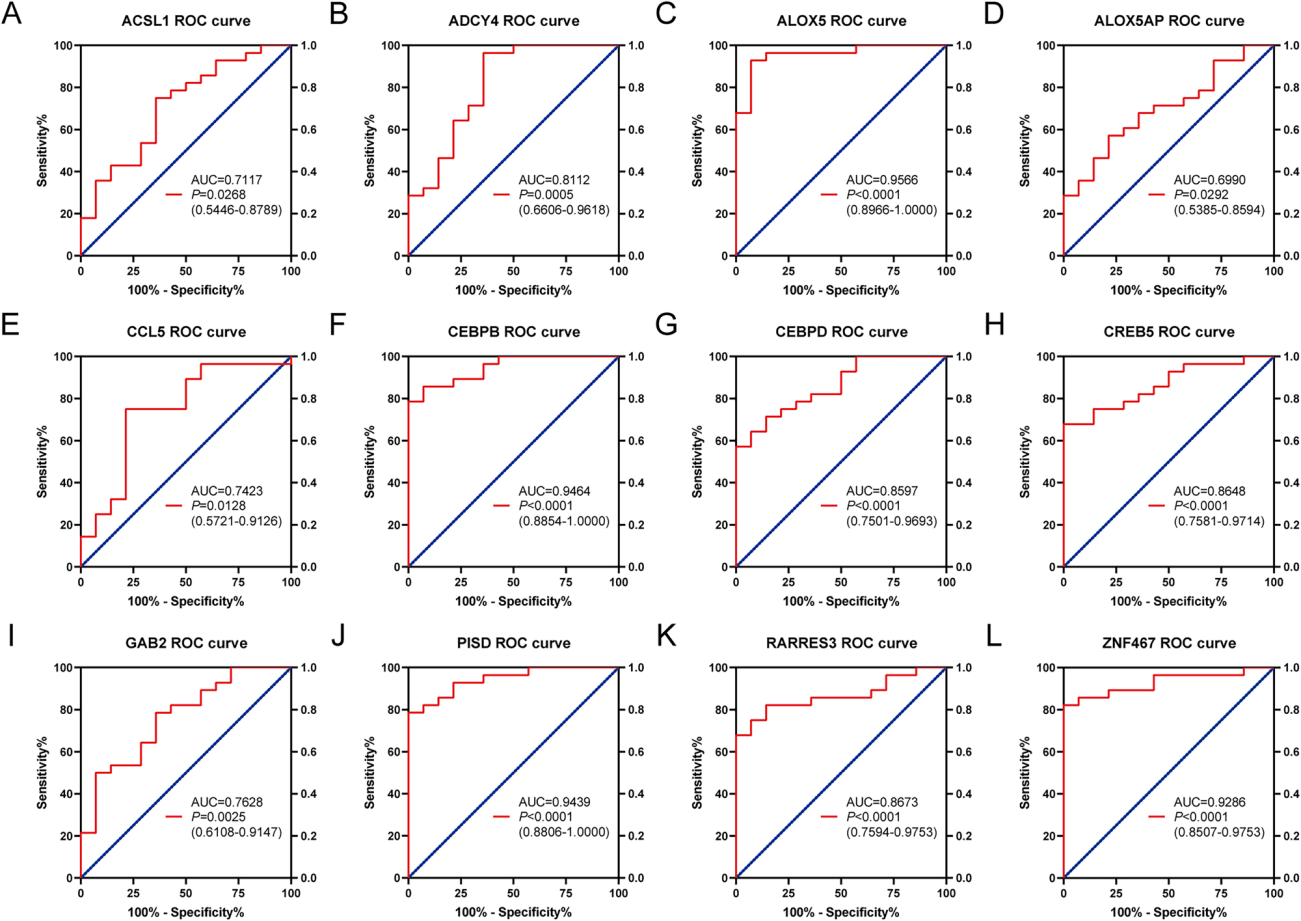
ROC curve analysis of Common differentially expressed LMRGs in external dataset GSE62646. (**A**–**L**) ROC curve analysis of *ACSL1*, *ADCY4*, *ALOX5*, *ALOX5AP*, *CCL5*, *CEBPB*, *CEBPD*, *CREB5*, *GAB2*, *PISD*, *RARRES3*, and *ZNF467* in dataset GSE62646.

ROC curve analysis was performed on these 12 significant differentially expressed LMRGs in GSE62646. Finally, the AUC of these 12 LMRGs were all ≥ 0.6, and 11 of them: *ACSL1* (AUC = 0.7117), *ADCY4* (AUC = 0.8112), *ALOX5*, (AUC = 0.9566), *CCL5* (AUC = 0.7423), *CEBPB* (AUC = 0.9464), *CEBPD* (AUC = 0.8592), *CREB5* (AUC = 0.8648), *GAB2* (AUC = 0.7628), *PISD* (AUC = 0.9439), *RARRES3* (AUC = 0.8673), and *ZNF467* (AUC = 0.9286), with AUC > 0.7 were identified as new potential biomarkers of AMI. Figure 5A–L presents the detailed results of the ROC analysis.

Sensitivity, specificity, and accuracy were calculated based on gene expression data. As shown in Table 1, *ALOX5*, *CEBPB*, *PISD*, and *ZNF467* have high sensitivity, specificity, and accuracy.

**Table 1:** The sensitivity, specificity, and accuracy of Common differentially expressed LMRGs for AMI.

Gene	True positive	True negative	Sensitivity	Specificity	Accuracy
ACSL1	22	8	0.79	0.57	0.71
ADCY4	23	9	0.82	0.64	0.76
ALOX5	26	12	0.93	0.86	0.90
ALOX5AP	20	6	0.71	0.43	0.62
CCL5	21	7	0.75	0.50	0.67
CEBPB	25	11	0.89	0.79	0.86
CEBPD	23	9	0.82	0.64	0.76
CREB5	23	9	0.82	0.64	0.76
GAB2	22	8	0.79	0.57	0.71
PISD	25	11	0.89	0.79	0.86
RARRES3	23	9	0.82	0.64	0.76
ZNF467	25	11	0.89	0.79	0.86

### Characteristics of immune infiltration in patients with *AMI*

**Figure 6:**
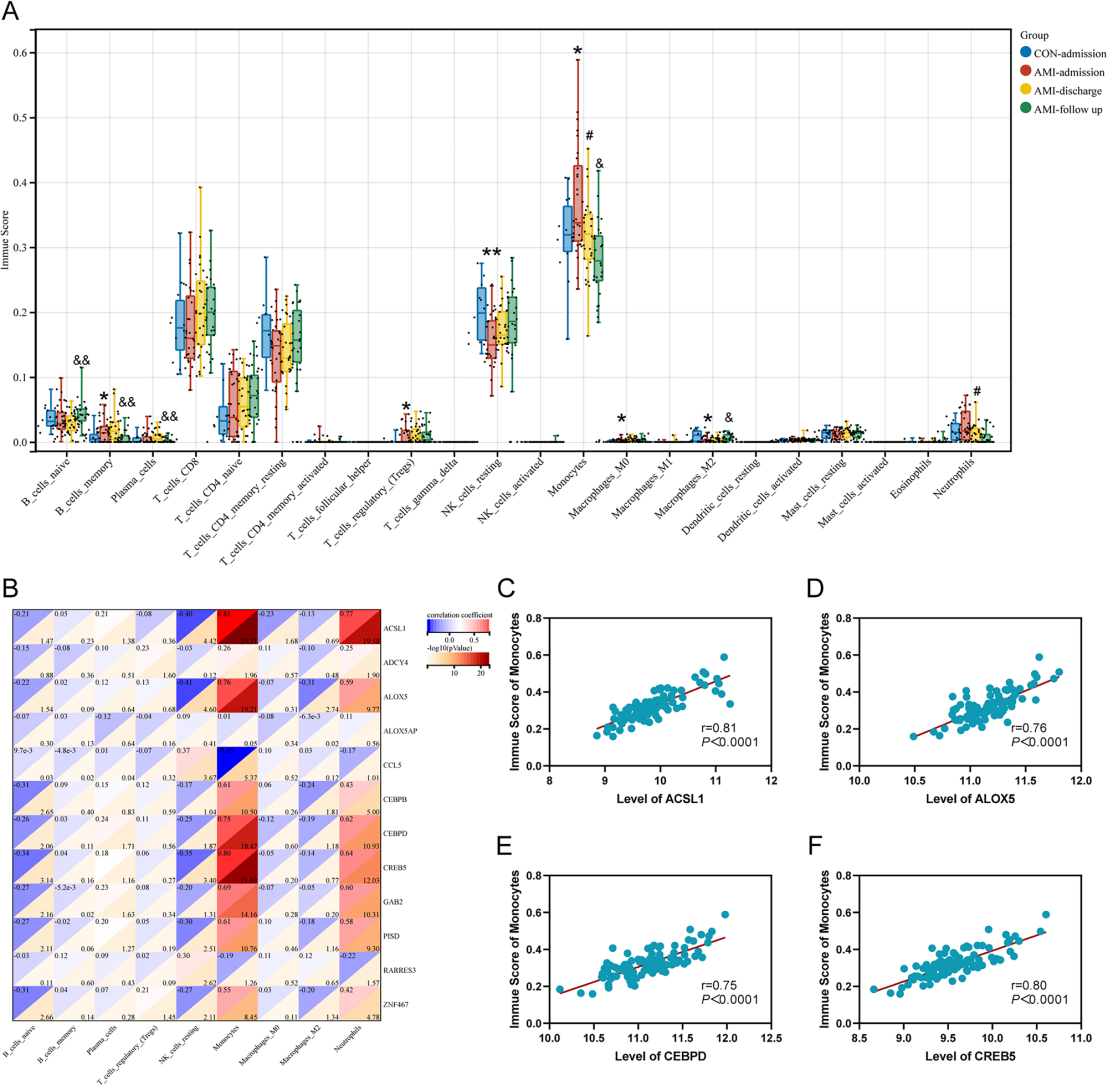
Characteristics of immune infiltration in patients with AMI. (**A**) The boxplot of 22 immune cell subtypes in AMI patients. (**B**) Correlation heatmap showing the correlation between LMRGs and immune cells. (**C**–**F**) The scatter plots showing the correlation between *ACSL1*, *ALOX5*, *CEBPD*, *CREB5* and monocytes. For panels (**A**) *AMI on admission (1st day of MI) vs Control; # AMI at discharge (4–6 days after MI) vs Control; & AMI at follow-up (6 months after MI) vs Control; *P < 0.05; **P < 0.01; ^#^P < 0.05; ^&^P < 0.05; ^&&^P < 0.01.

Immune infiltration analysis based on GSE62646 was performed to identify immune regulation in AMI. The results showed that AMI patients had a higher level of memory B cells, regulatory T cells (Tregs), Monocytes, M0 macrophages, and a lower level of resting NK cells, M2 macrophages (Fig. 6A). The Monocytes and Neutrophils of the AMI patients were significantly decreased after reperfusion therapy (Fig. 6A). In addition, Native B cells and M2 macrophages increased significantly, memory B cells, Plasma cells, and Monocytes decreased significantly 6 months after patient discharge (Fig. 6A). The Pearson correlation analysis was performed to explore the correlation between identified LMRGs and immune cells (Fig. 6B). The results showed that *ACSL1* (CRC = 0.81), *ALOX5* (CRC = 0.76), *CEBPD* (CRC = 0.75), and *CREB5* (CRC = 0.80) had a strong positive correlation with Monocytes (Fig. 6C–F).

### RT-qPCR verification of identified LMRGs in mouse *AMI* model

**Figure 7:**
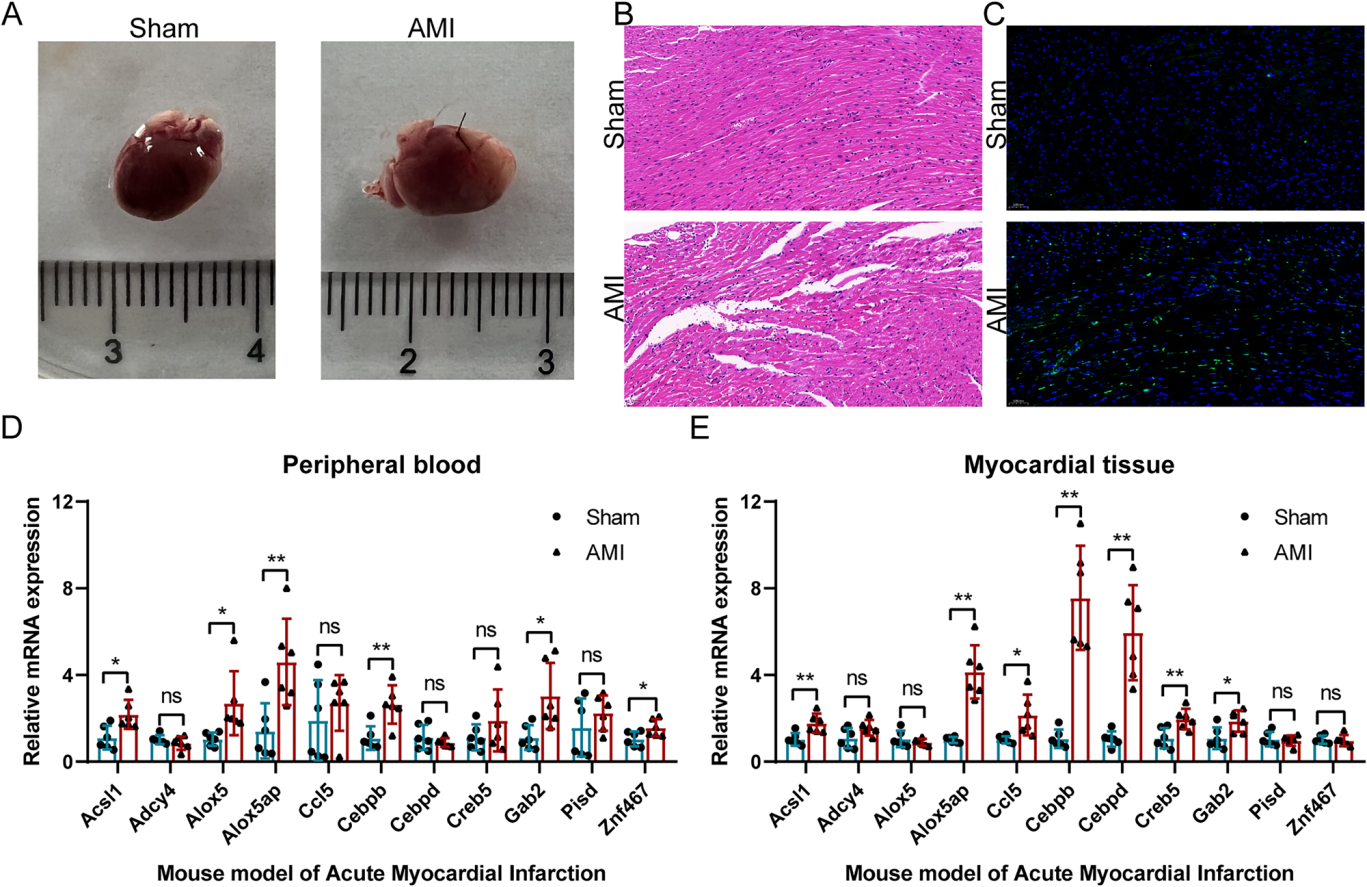
RT-qPCR verification of identified LMRGs in mouse AMI model. (**A**) Establishment of AMI model in mice. (**B**) HE staining results. (**C**) Tunel staining results. (**D**) Expression trends of *Acsl1*, *Adcy4*, *Alox5*, *Alox5ap*, *Ccl5*, *Cebpb*, *Cebpd*, *Creb5*, *Gab2*, *Pisd*, and *Znf467* in peripheral blood of mice with AMI (n Sham:n AMI = 6:6). (**E**) Expression trends of *Acsl1*, *Adcy4*, *Alox5*, *Alox5ap*, *Ccl5*, *Cebpb*, *Cebpd*, *Creb5*, *Gab2*, *Pisd*, and *Znf467* in myocardial tissue of mice with AMI (n Sham:n AMI = 6:6). For panels (**D**,**E**) ns P > 0.05; *P < 0.05; **P < 0.01.

To further validate the identified LMRGs, the mouse AMI model was generated (Fig. 7A). HE staining showed a disordered arrangement of myocardial cells and increased inflammatory cell infiltration in the infarcted myocardium (Fig. 7B). Tunel staining showed a significant increase in cell apoptosis during infarction (Fig. 7C). To verify the expression trend of the identified biomarkers (*Acsl1*, *Adcy4*, *Alox5*, *Alox5ap*, *Ccl5*, *Cebpb*, *Cebpd*, *Creb5*, *Gab2*, *Pisd*, and *Znf467*), RT-qPCR was performed on peripheral blood from mice with AMI (Fig. 7D). RT-qPCR results showed that 6 genes were significantly differentially expressed. They were *Acsl1*, *Alox5*, *Alox5ap*, *Cebpb*, *Gab2* and *Znf467*. Consistent with the analysis results, these genes were significantly upregulated in the blood of mice with AMI. To explore whether the identified genes are differentially expressed and play certain functions in cardiac tissue, we further performed RT-qPCR on the cardiac tissue of mice (Fig. 7E). The results showed that 7 genes were significantly differentially expressed. They were *Acsl1*, *Alox5ap*, *Ccl5*, *Cebpb*, *Cebpd*, *Creb5*, and *Gab2*. Notably, these genes were all significantly upregulated in myocardial tissue, while the trend of *Acsl1*, *Alox5ap*, *Cebpb*, and *Gab2* was consistent with the blood test results.

It should be especially mentioned that after careful searching of NCBI (https://www.ncbi.nlm.nih.gov/gene/?term=RARRES3) database and Biomart (https://bioconductor.org/packages/release/bioc/html/biomaRt.html) database, no homologous gene of *RARRES3* gene in the species of Mus musculus was found, so it was not tested.

## Discussion

AMI is one of the leading causes of cardiovascular death worldwide^14^. Since dead cardiomyocytes are unable to regenerate and repair, early reperfusion treatment has become the main measure to reduce myocardial infarction size, reduce mortality, and improve the prognosis of these patients^15^. Therefore, it is helpful and urgent to find biomarkers with high sensitivity and specificity for the early diagnosis of AMI to improve the clinical outcome of patients. Energy metabolism and inflammatory reactions play important roles in the formation and development of AMI, and lipid metabolism plays a vital part in the energy metabolism of myocardial cells^16^. Our study is based on the studies of lipid metabolism and its link with immunity in the field of AMI.

This study identified 23 common differentially expressed LMRGs in the existing AMI-related public datasets GSE61144 and GSE60993 in the GEO database. These genes had the same expression trends in both datasets. The results of enrichment analysis indicated that these genes were mainly associated with “lipid homeostasis”, “leukotriene production involved in inflammatory response”, “lipoxygenase pathway”, “metabolic pathways”, “regulation of lipolysis in adipocytes”, “insulin resistance”, “*TNF* signaling pathway” pathways. The String database was used for PPI analysis and a network diagram was constructed. It has been reported that adrenergic overdrive and adipose tissue lipolysis during AMI induce cardiac *AMPK*-*FGF21* feed-forward loop that may provide cardiac protection against ischemic injury^17^. The lipoxygenase pathway and its products 5-oxo-6,8,11,14-eicosatetraenoic acid (5-oxo-ETE) and leukotrienes induce cardiomyocyte injury in the mice model of ischemic myocardial injury and hypoxia/glucose deprivation^18^. The role of insulin resistance and the TNF signaling pathway in AMI has also been investigated^19–21^. These results suggest that the biological processes or signaling pathways predicted in this study are consistent with the existing findings. The mechanism of the identified LMRGs in the corresponding signaling pathways still needs to be further studied.

The external dataset GSE62646 further confirmed the differential expression of these 12 common differentially expressed LMRGs: *ACSL1*, *ADCY4*, *ALOX5*, *ALOX5AP*, *CCL5*, *CEBPB*, *CEBPD*, *CREB5*, *GAB2*, *PISD*, *RARRES3*, and *ZNF467*. Except for *ALOX5AP* (AUC = 0.6990), the AUC of these 12 genes is all over 0.7. A clinical study involving 75 AMI patients and 70 healthy controls suggests that *ACSL1* is highly expressed in the peripheral blood of AMI patients and may serve as a molecular marker for assessing the risk of AMI^22^. The diagnostic value of *CCL5* and *CEBPB* in AMI has also been identified in other studies^23,24^. These studies suggest that the genes identified in our study may serve as potential biomarkers to provide early warning signals of AMI.

The results of immune infiltration analysis showed that AMI patients had a higher level of memory B cells, regulatory T cells (Tregs), Monocytes, M0 macrophages, and a lower level of resting NK cells, M2 macrophages. These results are consistent with the previously reported results of AMI-related immune infiltration analysis^25,26^. The association of these identified immune cells with AMI has been partially investigated. One study has shown that *CD4*^+^ *FoxP3*^+^ *CD73*^+^ regulatory T cells promote cardiac healing after myocardial infarction^27^, and another study has shown that M0 macrophages upregulate and participate in immune regulation in AMI^25^. Exosomes derived from regulatory T cells could ameliorate AMI by promoting M2 polarization of macrophages^28^. The Pearson correlation analysis suggested a positive correlation between *ACSL1*, *ALOX5*, *CEBPD*, *CREB5* and Monocytes. It has been shown that LPS could induce *ALOX5* promoter activation and promote 5-lipoxygenase expression in human monocytes^29^. It has also been found that *CEBPD* is highly expressed in *CD14(*+*)* monocytes from patients with primary Sjogren's syndrome, and is involved in the *TNF-α* signaling pathway through *NF-κB*^30^. Whether these identified immune cells or genes are involved in AMI via a similar mechanism requires further investigation.

In addition, the expression of *Acsl1*, *Alox5*, *Alox5ap*, *Cebpb*, *Gab2*, and *Znf467* was confirmed in the mouse AMI model. *ACSL1* is a key rate-limiting enzyme in the process of lipid metabolism that catalyzes the conversion of long-chain fatty acids to their active form acyl-CoAs for cellular lipid synthesis ^31^. *ACSL1* overexpression in hepatocytes has been reported to increase intracellular triglyceride levels by reducing fatty acid β-oxidation through the PPARγ pathway^32^. The decreased expression of *ACSL1* can alleviate hypoxia-induced injury of AC16 cardiomyocytes^33^. *ALOX5* belongs to a class of non-heme iron dioxygenases involved in the catalysis of leukotriene biosynthesis. Targeted inhibition of *ALOX5* can protect the heart from remodeling and heart failure stimulated by hypertension by disturbing the LLPS of *Runx2* in cardiomyocytes^34^. Pharmacological inhibition of *ALOX12*, a member of the same gene family, ameliorates myocardial ischemia–reperfusion injury in a variety of animals^35^. *ALOX5AP* acts by activating *ALOX5*. *CEBPB* is an important transcription factor that regulates the expression of genes related to immune and inflammatory responses, and it also plays an important role in lipogenesis, gluconeogenesis, liver regeneration, and hematopoiesis^36^. GAB2 acts downstream of a variety of membrane receptors, such as cytokines, growth factor receptors, antigens, and hormones, and regulates a variety of signaling pathways. A recent study has shown that the *Gab2-MALT1* axis regulates thrombotic inflammation and is associated with deep vein thrombosis^37^. *ZNF467* is a transcription factor that promotes adipocyte differentiation and inhibits osteoblast differentiation in the bone marrow^38^. *CEBPB*, *GAB2*, and *ZNF467* have not been studied in the field of AMI, which represents a new research direction.

To explore whether the identified genes are differentially expressed and play certain functions in cardiac tissue, we further performed RT-qPCR on the cardiac tissue of mice. Finally, the expression of *Acsl1*, *Alox5ap*, *Ccl5*, *Cebpb*, *Cebpd*, *Creb5*, and *Gab2* was confirmed. Taken together, we found that *ACSL1*, *ALOX5AP*, *CEBPB*, and *GAB2* are significantly upregulated in AMI patients' blood, the peripheral blood of AMI mice, and the myocardial tissue of AMI mice. These genes may be used as potential biomarkers of AMI in blood, and may also affect the pathophysiological process of AMI by regulating lipid metabolism in cardiac myocytes.

This study is based on database mining and preliminary experiments, which also have some limitations. Although the results of multiple datasets were combined to enhance the strength of the results, the total sample size was still limited. Enrichment analysis and PPI analysis have explored the potential mechanisms of the identified LMRGs, but further functional experiments are still lacking. It is necessary to further explore the expression patterns of the identified genes in large human populations and conduct functional studies in vivo or in vitro to explore the exact mechanisms of these genes.

## Materials and methods

### Data acquisition and collation

Data mining of transcriptome sequencing data collected in online databases and finding target genes of interest is an important method for life science research. The AMI-related mRNA microarray datasets GSE61144, GSE60993, and GSE62646 were obtained from the Gene Expression Omnibus (GEO) database (https://www.ncbi.nlm.nih.gov/). These datasets all contain the following features: (1) From patients with AMI; (2) The sample source was human peripheral blood; (3) Provide complete raw mRNA profiling data. GSE61144 was obtained from the GPL6106 platform Sentrix Human-6 v2 Expression BeadChip. GSE60993 was obtained from the GPL6884 platform Illumina HumanWG-6 v3.0 expression beadchip^39^. GSE62646 was obtained from the GPL6244 platform [HuGene-1_0-st] Affymetrix Human Gene 1.0 ST Array [transcript (gene) version]^40^. The sample source for GSE61144 and GSE60993 was whole peripheral blood from human AMI patients and controls. The samples in GSE62646 were obtained from peripheral blood mononuclear cells of human AMI patients and controls. GSE61144 included 10 healthy controls and 7 AMI patients. GSE60993 included 7 healthy controls and 7 AMI patients. GSE62646 included 14 healthy controls and 28 AMI patients. Blood samples of AMI patients in GSE62646 were collected from patients on admission (1st day of MI), at discharge (4–6 days after MI), and at follow-up (6 months after MI). Supplementary File 3 presents the details of the included datasets and patients. The original gene expression data for each sample in these datasets were downloaded, and then further analysis was performed using GSE61144 and GSE60993 as the analysis datasets and GSE62646 as the external validation dataset.

The data preprocessing method is as follows: (1) Download the original gene expression matrix file; (2) Probe names were converted to gene names using the annotated list of datasets in the GEO database; (3) Quality control: Unqualified microarray data were excluded, such as genes with very low expression; (4) The log2 transformed was used to normalize the data.

### Identification of DEGs and differentially expressed LMRGs in *AMI*

Differential expression analysis of datasets GSE61144 and GSE60993 was performed by the “limma package” of R software (version 4.0.1) to screen differentially expressed genes (DEGs) between AMI and healthy controls. Benjamini–Hochberg method was used to correct adj. P for potential false positive results. The threshold for DEGs was set as adj. *P* value < 0.05 and |logFC|≥ 0.8 (FC stands for Fold change, it refers to the ratio of the mean relative gene expression of the AMI group to the control group). Previous studies related to bioinformatics analysis of lipid metabolism were retrieved and integrated to obtain LMRGs^41–44^. 1454 LMRGs were finally sorted out as candidate genes for subsequent analysis (Supplementary File 4). Differentially expressed LMRGs in datasets GSE61144 and GSE60993 were identified by the online Venn diagram website (http://bioinformatics.psb.ugent.be /webtools/Venn/). LMRGs that were differentially expressed in both datasets were considered as common differentially expressed LMRGs.

### Pathway enrichment analysis

Gene Ontology (GO) annotation and Kyoto Encyclopedia of Genes and Genomes (KEGG) pathway enrichment analysis^45^ were performed on the common differentially expressed LMRGs by the “cluster Profiler package” of R software (version 4.0.1) to explore the biological pathways and molecular mechanisms associated with these genes. GO annotation consists of biological process (BP) analysis, cellular component (CC) analysis, and molecular function (MF) analysis. A threshold of p < 0.05 was used to screen the relevant pathways. Based on the analysis results, the Sangerbox online website (version 3.0, http://sangerbox.com/home.html) was used to visualize and draw the relevant lollipop and chord diagrams^46^.

### Protein–protein interaction analysis

The String (https://cn.string-db.org) online database was used to carry out the protein–protein interaction (PPI) analysis to identify the interactions between the proteins expressed by the common differentially expressed LMRGs. The default parameters of the String database were used for analysis and the Cytoscape software was used to visualize the analysis results and draw the network diagram.

### Validation of common differentially expressed LMRGs in external datasets GSE62646

Dataset GSE62646 contains gene expression data on admission (1st day of MI), at discharge (4–6 days after MI), and at follow-up (6 months after MI) in AMI patients. Based on the original gene expression data in this dataset, the expression differences and dynamic changes of common differentially expressed LMRGs were further verified. Independent sample T-test was used for comparison between the two groups, and *P* < 0.05 was considered statistically significant.

### ROC curve analysis

Based on the original gene expression data of the AMI-admission group and control group in GSE62646 (log2 transformed data), the diagnostic value of the common differentially expressed LMRGs for AMI was assessed by receiver operating characteristic (ROC) curve analysis through GraphPad Prism software (version: 9.0). The area under the curve (AUC) ≥ 7.0 and *P* < 0.05 were used as screening criteria for potential biomarkers in this analysis. We then sorted the gene expression data in GSE62646 from small to large, and took the value ranked in 1/3 (high-expressed genes) as the diagnostic threshold of disease (nCON: nAMI = 1:2). For low-expressed genes, the value is sequenced in 2/3 digits. The sensitivity, specificity, and accuracy of each gene were calculated based on the diagnosis and actual results.

### Immune infiltration analysis

To further explore the association between LMRGs and immune response, the samples in GSE62646 were analyzed for immune infiltration by the CIBERSORT algorithm^47^. The composition and scores of 22 immune cell subpopulations in AMI and control groups were calculated by gene expression profiling. We performed log2 transformations on the original gene expression data and used the Pearson correlation analysis method to analyze the correlation between gene expression levels and immune cell scores. The “ggplot2 package” of R software was performed to visualize the analysis results.

### Establishment of the mouse *AMI* model

Animal facilities and experimental protocols were performed in accordance with the Care and Use of Laboratory Animals of the National Institute of Health (8th edition, 2011). C57BL/6 wild-type mice (SPF, male, 8-week-old) were purchased from the Shanghai Animal Laboratory Center. The mice were randomly divided into the sham operation group and the AMI group. The AMI model was established by ligation of the left anterior descending artery according to the previous published literature^48–50^. The brief method is as follows: after one week of adaptive rearing, mice were anesthetized with intraperitoneal injection of pentobarbital (70 mg/kg); After confirming no response to foot compression, endotracheal intubation was performed, the chest cavity was opened, and left anterior descending branch was ligated with 8.0 silk thread. To examine gene expression changes in the acute phase of AMI, heart tissue and orbital venous blood were obtained from mice at 24 h after AMI. This study was ethically reviewed and approved by the Animal Experiment Center of Wuhan University (Approval No. ZN2023201). All the experiments were conducted following the ARRIVE (Animal Research: Reporting of In Vivo Experiments) guidelines.

### Hematoxylin–eosin (HE) staining and TUNEL assay

To assess cardiac morphological features, freshly isolated hearts were fixed in 4% paraformaldehyde, embedded in paraffin, transverse sections (4–5 μm) were made and stained with HE. Images of stained cardiac tissue were obtained using a Leica DMI3000B microscope. To assess apoptosis, sections of paraffin-embedded myocardial tissue were stained using the TUNEL Kit (Roche Applied Science, Upper Bavaria, Germany). The results were observed by an inverted fluorescence microscope (Olympus, Tokyo, Japan), and the apoptotic cells were stained brown-yellow.

### Real-time quantitative polymerase chain reaction (RT-qPCR)

The expression of identified LMRGs was verified in the mouse AMI model by RT-qPCR. Total RNA extraction and reverse transcription of myocardial tissue and blood samples were performed by FastPure® Cell/Tissue Total RNA Isolation Kit V2 (Vazyme, Nanjing, China) and Hifair® III 1st Strand cDNA Synthesis SuperMix for qPCR (YEASEN, Shanghai, China). The Bio-Rad CFX96 Real-time PCR Detection System was performed to carry out the reaction, using Hieff UNICON® Universal Blue qPCR SYBR Green Master Mix (YEASEN, Shanghai, China). The reaction condition of the RT-qPCR reaction was set as (1) predenaturation 95 °C, 2 min. (2) denaturation 95 °C, 10 s. (3) Annealing/Extension 60 °C, 30 s. (4) Repeat 40 cycles in 2–3 steps. With Gapdh as the internal reference gene, the method of 2^−ΔΔCt^ was used to compare the expression changes of LMRGs between the mouse AMI group and the Sham group^51,52^. Supplementary File 5 presents the details of the primers used for the RT-qPCR reactions.

### Data analysis

Data analysis was performed using R (version 4.0.1) and GraphPad Prism (version 9.0) software. Data were presented as mean values ± standard error of the mean (SEM) from at least three independent experiments. Independent sample T-test was used for comparison between the two unpaired groups. *P* < 0.05 was considered statistically significant.

### Approval for animal experiments

All animal research and experiments in this study have been ethically reviewed and approved by the Animal Experiment Center of Wuhan University (Approval No. ZN2023201). The study was conducted in accordance with the local legislation and institutional requirements. All the experiments were conducted following the ARRIVE (Animal Research: Reporting of In Vivo Experiments) guidelines.

### Online Database address

Gene Expression Omnibus (GEO) database (https://www.ncbi.nlm.nih.gov/). Venn diagram website (http://bioinformatics.psb.ugent.be/webtools/Venn/). String database (https://cn.string-db.org). NCBI database (https://www.ncbi.nlm.nih.gov/gene/?term=RARRES3). Biomart database (https://bioconductor.org/packages/release/bioc/html/biomaRt.html). Sangerbox website (http://sangerbox.com/home.html).

## Conclusions

In this study, the AMI-related transcriptome microarray datasets were analyzed by bioinformatics methods, and the molecular mechanism of LMRGs involved in AMI was preliminarily explored. LMRGs *ACSL1*, *ALOX5AP*, *CEBPB*, and *GAB2* which are significantly associated with AMI were screened out. They are new potential biomarkers for AMI. In addition, the relationship between LMRGs and immune response has also been explored. This study provides new clues for the mechanism of AMI and provides a new direction for its clinical diagnosis and treatment.

### Supplementary Information

Supplementary Information 1.

Supplementary Information 2.

Supplementary Information 3.

Supplementary Information 4.

Supplementary Information 5.

Supplementary Information 6.

## Data Availability

The datasets presented in this study (GSE61144, GSE60993, and GSE62646) can be found in the GEO database (https://www.ncbi.nlm.nih.gov/).

## Abbreviations

AMI: Acute myocardial infarction
AUC: Area under the curve
BP: Biological process
CC: Cellular component
CRC: Correlation coefficient
DEG: Differentially expressed genes
GO: Gene Ontology
KEGG: Kyoto Encyclopedia of Genes and Genomes
LMRG: Lipid metabolism-related gene
MF: Molecular function
PCI: Percutaneous coronary intervention
PPI: Protein–protein interaction
ROC: Receiver operating characteristic
