# Supplementary material for: Expression characteristics of lipid metabolism-related genes and correlative immune infiltration landscape in acute myocardial infarction
Source: Sci Rep. 2024 Jun 18;14:14095. doi: 10.1038/s41598-024-65022-3 (PMC11189450; doi:10.1038/s41598-024-65022-3)
Supplement: Supplementary file 6 — Supplementary Information 6. [file 41598_2024_65022_MOESM6_ESM.docx]

**Supplementary table legends:**

**Supplementary File 1,** The details of the differentially expressed genes in GSE61144 and GSE60993.

**Supplementary File 2,** Detailed Pearson correlation analysis results of genes with strong correlations.

**Supplementary File 3,** The information of the included datasets and patients.

**Supplementary File 4,** Candidate LMRGs for subsequent analysis.

**Supplementary File 5,** The primer sequence information of the RT-qPCR experiment.
